# Treatment of Advanced Hepatocellular Carcinoma after Failure of Sorafenib Treatment: Subsequent or Additional Treatment Interventions Contribute to Prolonged Survival Postprogression

**DOI:** 10.1155/2017/5728946

**Published:** 2017-06-22

**Authors:** Masaaki Kondo, Kazushi Numata, Koji Hara, Akito Nozaki, Hiroyuki Fukuda, Makoto Chuma, Shin Maeda, Katsuaki Tanaka

**Affiliations:** ^1^Gastroenterological Center, Yokohama City University Medical Center, 4-57 Urafune-cho, Minami-ku, Yokohama, Kanagawa 232-0024, Japan; ^2^Department of Gastroenterology, Yokohama City University Graduate School of Medicine, 3-9 Fukuura, Kanazawa-ku, Yokohama, Kanagawa 236-0004, Japan

## Abstract

**Background:**

Sorafenib is a first-line treatment option for advanced hepatocellular carcinoma (HCC) patients; however, survival predictors upon progression have not been well characterized. In the present study, we aimed to show the efficacy of multidisciplinary therapy for patients who had failed to respond to sorafenib treatment.

**Methods:**

Among 146 BCLC stage B or C HCC patients treated with sorafenib monotherapy between July 2009 and August 2014, the first radiological progression according to the modified RECIST was identified in 71 patients; factors predicting overall survival (OS) and survival postprogression (SPP) were analyzed in these patients.

**Results:**

The median OS and SPP for patients who failed to respond to sorafenib treatment were 10.5 and 6.2 months, respectively, and the SPP was strongly correlated with the OS (*r* = 0.982, *P* < 0.01, and *R*^2^ = 0.965). The independent predictors of OS and SPP were identical. The predictors of SPP were des-gamma-carboxy prothrombin, progression of portal vein thrombosis, and subsequent second-line or additional treatment.

**Conclusions:**

SPP is closely associated with OS and might be notable in patients who have failed to respond to initial sorafenib treatment. Furthermore, interventions consisting of other treatment options upon the appearance of progression might prolong OS.

## 1. Introduction

Hepatocellular carcinoma (HCC) is one of the leading causes of cancer-related mortality globally [1]. According to the Barcelona clinic liver cancer (BCLC) staging system, stage B patients with continuous progression after transarterial chemoembolization (TACE) and stage C patients have an exceedingly poor prognosis [2, 3]. Sorafenib, a multikinase inhibitor, is the only proven global standard treatment for BCLC stage C and is also recommended for TACE-refractory patients with stage B disease [4]. Sorafenib has been reported to improve the overall survival (OS) of advanced HCC patients, compared with those receiving a placebo [5, 6] and is currently the only first-line treatment available for BCLC stage C patients. Although about half of these patients achieve disease control, a critical issue in clinical practice is the absence of a second-line treatment for those who fail to respond to sorafenib treatment. The rapid in vivo vascular regrowth of tumors occurs as a result of the reversal of vascular endothelial growth factor (VEGF) inhibition [7], so the discontinuation of sorafenib after progressive disease (PD) might actually lead to more rapid progression. The administration of sorafenib after first PD in patients with extrahepatic metastasis, rather than terminating treatment at the time of PD, could provide continuous HCC growth suppression, potentially prolonging survival [8]. In clinical practice, other alternative options, including hepatic arterial infusion chemotherapy (HAIC), are often administered as a second-line treatment option for patients who have failed to respond to sorafenib treatment [9, 10]. Recently, Tanaka et al. [11] reported that most long-term survivors with more than 3 years of survival after initial sorafenib treatment had received other treatment modalities in the form of multidisciplinary therapy. However, the efficacy of interventions using other treatment options upon progression has not been well defined. In this study, we investigated whether the interventions of subsequent second-line or additional treatment options could contribute to survival postprogression (SPP) for patients who failed to respond to initial sorafenib treatment.

## 2. Patients and Methods

### 2.1. Patients

**Figure 1:**
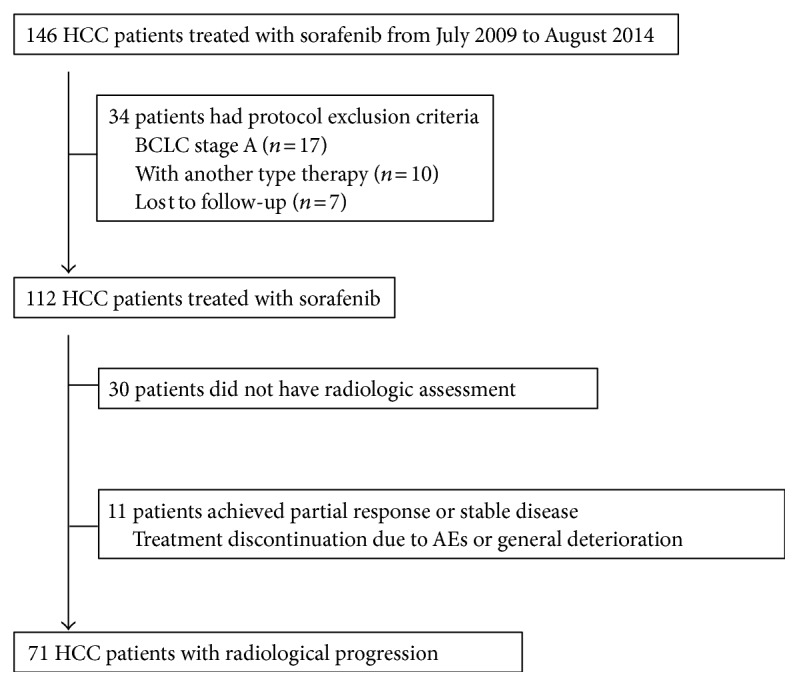
Enrollment scheme and outcomes of selected patients with progressive disease among patients with hepatocellular carcinoma who failed to respond to initial sorafenib treatment.

We enrolled 146 consecutive patients who had received sorafenib treatment between July 2009 and August 2014 at Yokohama City University Medical Center, and those with progressive HCC were radiologically evaluated. HCC was diagnosed based on pathological findings or radiological dynamic studies according to the criteria of the American Association for the Study of Liver Diseases [4]. The eligibility criteria for this study were as follows: (1) a tumor stage equivalent to BCLC stage B or C and (2) a tumor response assessed as PD based on radiological dynamic studies according to the modified response evaluation criteria in solid tumors (mRECIST) [12]. The exclusion criteria were as follows: (1) BCLC stage A, (2) sorafenib treatment with concomitant therapy, and (3) an inability to complete the follow-up schedule. Among the 146 HCC patients who were initially enrolled, 34 patients were subsequently excluded for the following reasons: BCLC stage A (*n* = 17), other concomitant therapy (*n* = 10), and lost to follow-up (*n* = 7). Among the 112 eligible patients, 71 patients exhibited progression radiologically (Figure 1). This study was approved by the Institutional Review Board of the Yokohama City University Medical Center. The informed consent requirement was waived for this analysis.

### 2.2. Sorafenib Treatment and Evaluation of Its Toxicity and Efficacy

An initial full-dose regimen of sorafenib was 400 mg administered twice daily. An initial dose reduction to 400 mg daily was allowed for some patients, including the elderly or those with poor liver function. Treatment interruptions and dose reductions were also allowed if any of the patients experienced severe adverse events. The toxicities of the sorafenib treatment were evaluated based on the Common Terminology Criteria for Adverse Events (CTCAE 3.0) [13]. The clinical response was assessed according to the mRECIST criteria [12], based on dynamic computed tomography (CT) or magnetic resonance imaging (MRI) results obtained every 6 to 8 weeks during treatment. We assessed the radiological progression of intrahepatic or extrahepatic growth corresponding to a more than 20% increase in the target lesion against the baseline or progression of nontarget intrahepatic or extrahepatic lesions, the appearance of a new intrahepatic or extrahepatic lesion, or the appearance or extension of macrovascular invasion.

### 2.3. Treatment Options for Postradiological PD

At the first evidence of radiological PD after sorafenib treatment, an attending physician decided whether to continue sorafenib treatment and whether subsequent or additional treatment options should be administered. The treatment options were classified into two categories: subsequent treatment, corresponding to the termination of sorafenib treatment and the administration of another treatment and additional treatment, corresponding to the continuation of sorafenib treatment with the addition of another treatment. Multidisciplinary treatments included sorafenib continuation, and other subsequent or additional second-line treatment options included TACE, HAIC, radiotherapy, high-intensity focused ultrasound (HIFU), or clinical trials involving the following drugs: everolimus, tivantinib, regorafenib, brivanib, or a combination of tegafur, gimeracil, and oteracil potassium.

The treatment scheme after radiological PD was divided into five groups: group 1—subsequent second-line treatments were administered with the interposition of sorafenib continuation; group 2—additional treatments were administered with sorafenib continuation; group 3—subsequent second-line treatment without the continuation of sorafenib; group 4—best supportive care was administered with the interposition of sorafenib continuation; and group 5—best supportive care without the continuation of sorafenib.

### 2.4. Statistical Analysis

Survival data (OS, SPP, and time to progression (TTP)) and correlations among these indices were evaluated for the treatment groups. OS was defined as the time from the start of sorafenib treatment until death from any cause or the last medical examination. SPP was defined as the time from first evidence of radiological PD after sorafenib treatment until death from any cause or the last medical examination. TTP was defined as the time from the start of sorafenib treatment until radiological PD. Statistical analyses were performed using SPSS 21.0 software (SPSS Inc., Chicago, IL, USA). Continuous parameters were expressed as medians and ranges, and categorical variables were expressed as numbers and percentages or frequencies. The clinical characteristics of the subgroups were compared using the Kruskal-Wallis test. A Spearman rank correlation analysis and a linear regression analysis were used to examine the correlations among OS, SPP, and TTP. The survival curves were plotted using Kaplan-Meier methods, and significant differences between two groups were compared using the log-rank test. Variables with a *P* value of less than 0.05 were regarded as significant in the univariate analysis and were included in the multivariate analysis. The Cox proportional hazard regression was used to assess primary factors associated with OS and SPP.

## 3. Results

### 3.1. Patient Characteristics and Therapeutic Responses to Sorafenib

Table 1 shows the baseline characteristics of the patients at the start of the sorafenib treatment. Twenty-nine patients (40.8%) received 400 mg twice daily, while 42 patients initially received less than 800 mg daily. The median duration of therapy was 4.3 months (range, 0.1–41.1 months). Five (7%) patients exhibited a partial response (PR), 22 (31%) had stable disease (SD), and 44 (62%) had PD. The median OS and SPP were 10.5 (1.0–71.1) and 6.2 (0.4–65.3) months, respectively. Adverse events of grade 3 or 4 were observed in 40 patients (56.3%). Fifteen patients (21.1%) discontinued sorafenib treatment because of unacceptable drug-related toxicities. The most commonly observed adverse events of grade 3 or higher were hand-foot skin reaction (20%), an elevated aspartate aminotransferase level (15%), and an elevated alanine aminotransferase level (11%).

### 3.2. Treatment Schemes after Radiological PD in Patients Treated with Sorafenib

**Figure 2:**
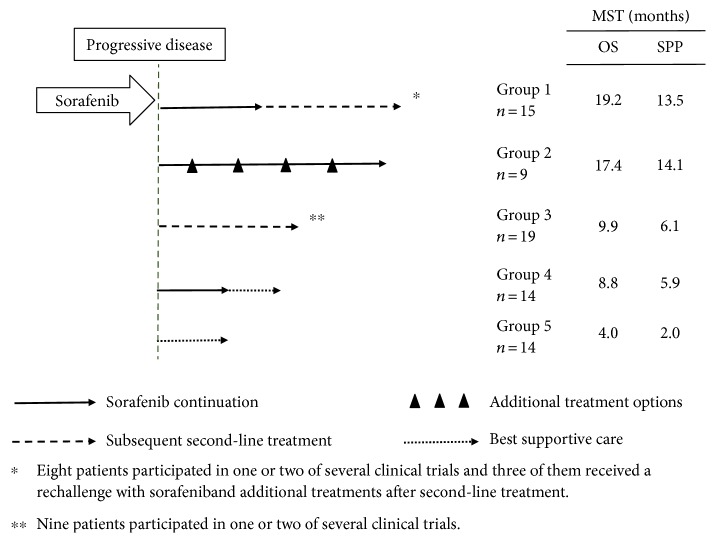
Treatment scheme after radiological progression of sorafenib treatment. Patients were subdivided according to whether or not sorafenib treatment was continued and whether or not subsequent second-line or additional treatments were administered.

All the treatment decisions were made at the discretion of the treating physicians, and the treatment schemes administered after radiological PD were classified into five patterns (Figure 2), subdivided according to whether or not sorafenib treatment was continued and whether or not subsequent second-line or additional treatments were administered. Sorafenib treatment was continued in 15 patients (group 1) but was not continued in the remaining 19 patients (group 3). Subsequent treatment options included TACE (*n* = 10), HAIC (*n* = 14), a combination of tegafur, gimeracil, and oteracil potassium (*n* = 3), and clinical placebo-controlled randomized trials (*n* = 19) that included everolimus (*n* = 3), tivantinib (*n* = 2), regorafenib (*n* = 2), brivanib (*n* = 2), or a combination of tegafur, gimeracil, and oteracil potassium (*n* = 10) after sorafenib failure. Nineteen clinical trials were administered in 17 patients. Fourteen of these patients received the active drugs and 2 of these patients received the active drugs in 2 separate clinical trials. However, the remaining 3 patients received the placebo. Two of these patients had participated in a trial examining a combination of tegafur, gimeracil, and oteracil potassium and received combination treatments of sorafenib rechallenge with TACE or HAIC after the trials (group 1), while one patient in a trial examining everolimus received HAIC after the trial (group 3). In group 1, one patient underwent sorafenib rechallenge with additional TACE and HIFU after participating in a clinical trial with tivantinib as positive control. Additional treatment options included TACE (*n* = 8), radiotherapy (*n* = 3), and HAIC (*n* = 3) in 9 patients with sorafenib continuation (group 2). No subsequent second-line or additional treatment options other than sorafenib were administered in 28 patients (best supportive care); sorafenib was continued after radiological PD in 14 patients (group 4) but was not continued in the remaining 14 patients (group 5). The median OS and SPP were longest for groups 1 and 2, followed by groups 3 and 4, and were shortest for group 5.

Table 2 shows the patient characteristics at the time of radiological PD after sorafenib treatment in each of the treatment groups shown in Figure 2. No significant differences in the Child-Pugh class and BCLC stage were observed among the groups. However, significant differences in the serum AST, albumin levels, and the frequency of progressive macrovascular invasion were observed among the groups; the last index were prominent in group 5.

### 3.3. Predictors of OS and SPP

**Figure 3:**
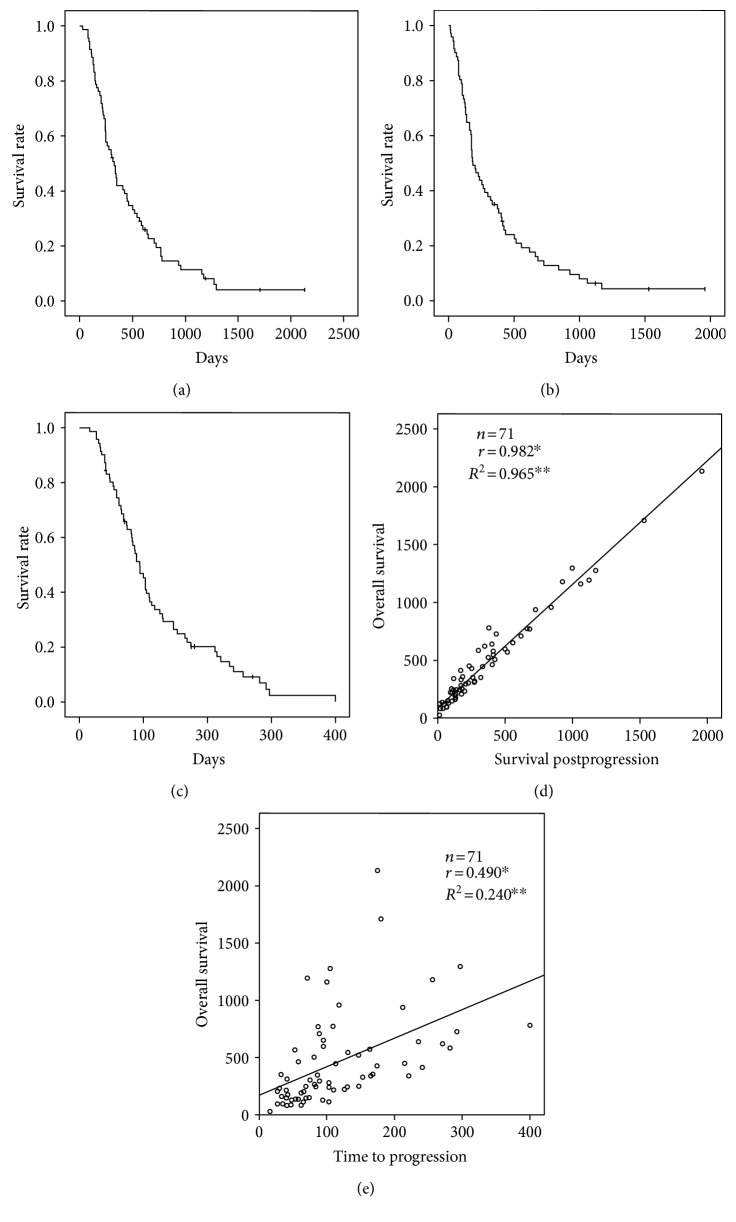
Kaplan-Meier survival curves for hepatocellular carcinoma patients receiving sorafenib treatment and correlations among overall survival, survival postprogression, and time to progression. (a) Overall survival. (b) Survival postprogression after first progression of sorafenib treatment. (c) Time to progression. (d) Correlation between overall survival and survival postprogression. (e) Correlation between overall survival and time to progression. ^∗^The *r* values represent Spearman's rank correlation coefficient. ^∗∗^The *R*^2^ values represent the linear regression.

The median OS, SPP, and TTP for patients who failed to respond to sorafenib treatment were 10.5, 6.2, and 3.0 months, respectively (Figures 3(a)–3(c)). SPP was strongly correlated with OS (*r* = 0.982, *P* < 0.01, and *R*^2^ = 0.965), but TTP was weakly correlated with OS (*r* = 0.490, *P* < 0.01, and *R*^2^ = 0.240) (Figures 3(d)‐3(e)).

Tables 3 and 4 show the predictors for OS and SPP in the 71 patients with radiological progression based on univariate and multivariate analyses. The baseline predictors in the univariate analysis of OS were alpha-fetoprotein (AFP) (*P* = 0.026), des-gamma-carboxy prothrombin (DCP) (*P* < 0.001), subsequent second-line or additional treatment (*P* = 0.026), progression of macrovascular invasion (*P* < 0.001), sorafenib treatment for more than 3 months before radiological PD (*P* = 0.008), and sorafenib continuation after radiological PD (*P* = 0.048). In the multivariate analysis, DCP (*P* < 0.001), subsequent second-line or additional treatment (*P* = 0.013), and progression of macrovascular invasion (*P* = 0.003) were independently associated with OS (Table 3).

The baseline predictors in the univariate analysis of SPP were AFP (*P* = 0.022), DCP (*P* < 0.001), subsequent second-line or additional treatment (*P* = 0.037), and progression of macrovascular invasion (*P* = 0.001). Sorafenib continuation after radiological PD had a tendency to provide a survival benefit (*P* = 0.050), but the difference was not significant in the univariate analysis. In the multivariate analysis, DCP (*P* < 0.001), subsequent second-line or additional treatment (*P* = 0.019), and progression of macrovascular invasion (*P* = 0.035) were independently associated with SPP (Table 4).

## 4. Discussion

To obtain long-term survival after sorafenib treatment, various treatment modalities other than sorafenib are often administered in the form of multidisciplinary therapy [11]; however, the efficacy of multidisciplinary treatment in patients who have failed to respond to sorafenib has not yet been established. In the present study, we showed for the first time that interventions consisting of subsequent second-line or additional treatment options at the time of the radiological progression after sorafenib treatment contributed to a longer OS and SPP for HCC patients with BCLC stage B or C disease.

HCC patients might exhibit molecular heterogeneity [14, 15] and a nonuniform baseline population. Therefore, multidisciplinary therapy might have some impact on the treatment of HCC patients with various stages of disease. In a review article summarizing data from eight Japanese institutions, various salvage options during sorafenib treatment or post-sorafenib therapy had been administered to long-term survivors of over three years [11]. In the present study, additional local treatment (TACE or HAIC) or subsequent second-line targeted therapy were administered in 43 patients (61%), contributing to the elongation of the survival period after the radiological progression of sorafenib treatment in these patients.

Additional local treatment options might be beneficial for controlling the liver tumor burden in patients with metastasized HCC [16]. However, a prospective randomized phase 3 trial conducted in Japan and Korea failed to show a combined effect of TACE and sorafenib [17]. Thereafter, a survival advantage of TACE combined with sorafenib was suggested over TACE alone, retrospectively [18], and over sorafenib alone in the final analysis of the GIDEON study [19]. Regarding another sequential second-line or additional option, HAIC has been frequently administered to HCC patients who fail to respond to sorafenib, and the expected efficacy and tolerability were reported [9, 10]. The disease control rates were both over 60% and no intolerable adverse events were reported. Recently, the results of two randomized controlled studies to evaluate the additional effect of HAIC were reported. The HAIC regimens in these trials include low-dose CDDP with 5-FU (SILIUS trial: NCT01214343) and CDDP powder (CDDP-Sor-rP2 study: UMIN000005703). In the SIRIUS trial, a combination treatment with sorafenib and HAIC did not improve the OS in advanced HCC patients, compared to that with sorafenib alone. However, in those with main portal vein trunk invasion, the combination treatment significantly improved the OS, compared to that with sorafenib alone [20]. On the other hand, a combination treatment with sorafenib and CDDP powder tended to improve the OS in advanced HCC patients, compared to that with sorafenib [21]. Regarding subsequent second-line molecular-targeted drugs, previous clinical trials have failed to show any efficacy against HCC among patients who have failed to respond to sorafenib [22, 23]. In the present study, some molecular-targeted drugs, such as brivanib, everolimus, tivantinib, and regorafenib, were administered as part of clinical trials after sorafenib failure (groups 1 and 3). Second-line clinical trials evaluating brivanib and everolimus failed to demonstrate a survival benefit [22, 23]; this may mimic the impact of subsequent second-line treatment options other than sorafenib continuation after first-line sorafenib failure. Recently, positive data from the RESORCE trial examining regorafenib were reported [24].

In the present study, the independent predictors of OS and SPP were identical in the multivariate analyses, and a strong correlation between OS and SPP was clarified, showing that SPP may be a potential key factor of OS among patients who have failed to respond to sorafenib treatment. SPP has been shown to be strongly correlated with OS in nonsmall cell lung cancer [25], gastric cancer [26], and HCC [27]; in these malignancies, the TTP or progression-free survival (PFS) becomes weaker as the proportion of SPP to OS increases. In contrast, the PFS or TTP is supposedly well correlated with OS in pancreatic cancer [28], metastatic colorectal cancer [29], and metastatic renal cell cancer [30], in which the SPP remains short because the advantage of second-line chemotherapy is limited. Therefore, the strong correlation between SPP and OS may support an impact beyond radiological PD treatment options after sorafenib treatment. Furthermore, a high DCP level and the progression of macrovascular invasion at the time of PD were associated with shorter OS and SPP. Regarding negative predictors observed in the present study, they may be compatible with those reported by Reig et al. [31]; the SPP was influenced by the progression patterns, especially in BCLC-C patients with new extrahepatic lesions and/or vascular invasion.

The present study had some limitations. First, the study had a relatively small sample size. Second, this study was performed retrospectively, so only patients who were expected to have a longer prognosis, whose liver functions were retained or whose disease conditions had not worsened, and who received subsequent or additional treatment, were included. However, this study suggested that HCC patients who had progressed radiologically after receiving sorafenib treatment were candidates for subsequent or additional treatment options in clinical practice. Third, the second-line treatment options were heterogeneous and complicated, especially for the continued use of sorafenib after radiological progression. To confirm the efficacy of continued sorafenib treatment after the radiologic detection of PD, further prospective studies involving a larger number of subjects may be required.

In conclusion, SPP was strongly correlated with OS among patients who failed to respond to initial sorafenib treatment, and interventions consisting of other treatment options in the form of multidisciplinary therapy upon progression may be useful for prolonging the SPP and OS.

## Figures and Tables

**Table 1:** Patient characteristics at the initiation of sorafenib treatment.

Characteristics	*n* = 71
Sex	
Male/female	54/17
Median age (years)	74 (48–88)
Cause	
HCV/HBV/others	48/8/15
TNM stage	
II/III/IVa/IVb	3/39/15/14
BCLC stage	
B/C	41/30
Child-Pugh class	
A/B/NA^∗^	59/11/1
Macrovascular invasion	
Yes/no	17/54
Extrahepatic metastasis	
Yes/no	14/57
ALT (U/L)^†^	37 (9–119)
AST (U/L)^†^	59 (14–268)
Total bilirubin (mg/dL)^†^	0.9 (0.5–2.6)
Albumin (g/dL)^†^	3.7 (2.4–4.9)
AFP (ng/mL)^†^	171 (2–589,420)
DCP (mAU/mL)^†^	313 (19–366,930)

^∗^The Child-Pugh status of one patient could not be classified because of warfarin usage for cardiovascular disease. ^†^Data are the median values. HCV: hepatitis C virus; HBV: hepatitis B virus; BCLC; Barcelona clinic liver cancer; ALT: alanine aminotransferase; AST: aspartate aminotransferase; AFP: alpha-fetoprotein; DCP: des-gamma carboxy-prothrombin.

**Table 2:** Patient characteristics of five groups classified according to treatment patterns after radiological progression of sorafenib treatment.

	Group 1 (*n* = 15)	Group 2 (*n* = 9)	Group 3 (*n* = 19)	Group 4 (*n* = 14)	Group 5 (*n* = 14)	*P* value
Sex (male/female)	10/5	7/2	15/4	10/4	12/2	0.793
Median age	74 (63–85)	72 (60–88)	72 (54–83)	74 (48–88)	79 (59–85)	0.324
Child-Pugh class (A/B)	14/1	9/0	16/2	10/4	10/4	0.178
BCLC stage (B/C)	11/4	6/3	11/8	6/8	7/7	0.500
AST (U/L)	40 (17–135)	42 (14–98)	77 (22–268)	65.5 (17–99)	67.5 (29–144)	0.042
ALT (U/L)	23 (10–119)	30 (9–83)	55 (15–93)	38.5 (15–94)	41.5 (14–63)	0.517
Albumin (g/dL)	3.7 (3.3-4.7)	4.0 (3.0–4.9)	3.6 (2.8–4.4)	3.5 (2.8–4.2)	3.4 (2.4–4.5)	0.017
Total bilirubin (mg/dL)	1.0 (0.5–2.2)	0.7 (0.6–0.9)	1.0 (0.5–1.7)	1.0 (0.5–2.6)	1.1 (0.6–2.1)	0.173
AFP (ng/mL)	33 (2–29,389)	129 (2–5967)	293.5 (4–55,298)	197.5 (5–14,634)	865.5 (4–589,420)	0.102
DCP (mAU/mL)	194 (22–5032)	140 (39–24,975)	1271 (21–325,960)	260 (47–19,584)	963.5 (19–366,930)	0.248
Best overall response						
PR+SD/PD	9/6	5/4	7/12	4/10	2/12	0.091
Progression of MVI (yes/no)	2/13	1/8	5/14	1/13	8/6	0.015
Intrahepatic growth (yes/no)	11/4	5/4	13/6	7/7	4/10	0.644
Extrahepatic growth (yes/no)	1/14	2/7	2/17	5/9	3/11	0.280
New intrahepatic lesion (yes/no)	7/8	3/6	5/14	3/11	5/9	0.641
New extrahepatic lesion (yes/no)	0/15	0/9	2/17	3/11	1/13	0.667
Treatment duration before PD						
≥3/<3 months	10/5	4/5	7/12	8/6	2/12	0.071

The patients were stratified into five groups according to treatment course after radiological progression as follows: group 1—subsequent second-line treatment options following sorafenib continuation; group 2—additional treatment options with sorafenib continuation; group 3—subsequent second-line treatment options; group 4—best supportive care following sorafenib continuation; group 5—best supportive care. BCLC: Barcelona clinic liver cancer; AST: aspartate aminotransferase; ALT: alanine aminotransferase; AFP: alpha-fetoprotein; DCP: des-gamma carboxy-prothrombin; PR: partial response; SD: stable disease; PD: progressive disease. Intrahepatic growth: intrahepatic increase of more than 20% of the target lesion in a previously documented lesion or the progression of a nontarget lesion; extrahepatic growth: extrahepatic increase of more than 20% of the target lesion in a previously documented lesion or the progression of a nontarget lesion; progression of MVI: appearance or extension of macrovascular invasion.

**Table 3:** Prognostic factors for overall survival based on univariate and multivariate analyses.

Variables		Univariate analysis	Multivariate analysis		
		*P* value	*P* value	HR	95% CI
Age	≥75 years	0.852			
	<75 years				
Sex	Male	0.518			
	Female				
Cause	HCV	0.711			
	Others				
Child-Pugh class	A	0.276			
	B				
BCLC stage	B	0.154			
	C				
AST (U/L)	<80	0.924			
	≥80				
ALT (U/L)	<80	0.834			
	≥80				
Total bilirubin (mg/dL)	<1.0	0.843			
	≥1.0				
Albumin (g/dL)	<3.6	0.149			
	≥3.6				
AFP (ng/mL)	≥400	0.026	0.971	0.990	0.568–1.726
	<400				
DCP (mAU/mL)	≥400	<0.001	<0.001	3.443	1.818–6.520
	<400				
Best overall response	PR+SD	0.052			
	PD				
Subsequent or additional treatment	Yes	0.026	0.013	0.499	0.288–0.865
	No				
Progression of macrovascular invasion	Yes	<0.001	0.003	2.974	1.453–6.088
	No				
Intrahepatic growth	Yes	0.577			
	No				
Extrahepatic growth	Yes	0.376			
	No				
New intrahepatic lesion	Yes	0.071			
	No				
New extrahepatic lesion	Yes	0.569			
	No				
Treatment duration before PD (months)	≥3	0.008	0.321	0.757	0.436–1.313
	<3				
Sorafenib continuation after PD	Yes	0.048	0.969	0.989	0.576–1.700
	No				

HCV: hepatitis C virus; BCLC: Barcelona clinic liver cancer; AST: aspartate aminotransferase; ALT: alanine aminotransferase; AFP: alpha-fetoprotein; DCP: des-gamma carboxy-prothrombin; PR: partial response; SD: stable disease; PD: progressive disease.

**Table 4:** Prognostic factors for survival postprogression based on univariate and multivariate analyses.

Variables		Univariate analysis	Multivariate analysis		
		*P* value	*P* value	HR	95% CI
Age	≥75 years	0.851			
	<75 years				
Sex	Male	0.491			
	Female				
Cause	HCV	0.572			
	Others				
Child-Pugh class	A	0.100			
	B				
BCLC stage	B	0.062			
	C				
AST (U/L)	<80	0.963			
	≥80				
ALT (U/L)	<80	0.879			
	≥80				
Total bilirubin (mg/dL)	<1.0	0.837			
	≥1.0				
Albumin (g/dL)	<3.6	0.098			
	≥3.6				
AFP (ng/mL)	≥400	0.022	0.864	1.050	0.603–1.828
	<400				
DCP (mAU/mL)	≥400	<0.001	<0.001	3.936	2.066–7.497
	<400				
Best overall response	PR+SD	0.317			
	PD				
Subsequent or additional treatment	Yes	0.037	0.019	0.529	0.310–0.900
	No				
Progression of macrovascular invasion	Yes	0.001	0.035	2.025	1.052–3.898
	No				
Intrahepatic growth	Yes	0.769			
	No				
Extrahepatic growth	Yes	0.244			
	No				
New intrahepatic lesion	Yes	0.081			
	No				
New extrahepatic lesion	Yes	0.217			
	No				
Treatment duration before PD (months)	≥3	0.093			
	<3				
Sorafenib continuation after PD	Yes	0.050			
	No				

HCV: hepatitis C virus; BCLC: Barcelona clinic liver cancer; AST: aspartate aminotransferase; ALT: alanine aminotransferase; AFP: alpha-fetoprotein; DCP: des-gamma carboxy-prothrombin; PR: partial response; SD: stable disease; PD: progressive disease.
